# Supplementary material for: A Critical View of the Application of the APEX Software (Aqueous Photochemistry of Environmentally-Occurring Xenobiotics) to Predict Photoreaction Kinetics in Surface Freshwaters
Source: Molecules. 2019 Dec 18;25(1):9. doi: 10.3390/molecules25010009 (PMC7017383; doi:10.3390/molecules25010009)
Supplement: Supplementary file 1 [file molecules-25-00009-s001.zip › 3.2.4_gcc-4.4.0.zip!/3.2.4_gcc-4.4.0/3.2.4_gcc-4.4.0/doc/octave/html/interpreter/Subfunctions.html]

Subfunctions - Untitled


Next: Private Functions,
Previous: Manipulating the load path,
Up: Function Files


---

#### 11.7.2 Subfunctions

A function file may contain secondary functions called
subfunctions. These secondary functions are only visible to the
other functions in the same function file. For example, a file
f.m containing

```
     function f ()
       printf ("in f, calling g\n");
       g ()
     endfunction
     function g ()
       printf ("in g, calling h\n");
       h ()
     endfunction
     function h ()
       printf ("in h\n")
     endfunction
```

defines a main function `f` and two subfunctions. The
subfunctions `g` and `h` may only be called from the main
function `f` or from the other subfunctions, but not from outside
the file f.m.
